# Assessment of Arbovirus Surveillance 13 Years after Introduction of West Nile Virus, United States^1^

**DOI:** 10.3201/eid2107.140858

**Published:** 2015-07

**Authors:** James L. Hadler, Dhara Patel, Roger S. Nasci, Lyle R. Petersen, James M. Hughes, Kristy Bradley, Paul Etkind, Lilly Kan, Jeffrey Engel

**Affiliations:** Yale University School of Public Health, New Haven, Connecticut, USA (J.L. Hadler);; Council of State and Territorial Epidemiologists, Atlanta, Georgia, USA (D. Patel, J. Engel);; Centers for Disease Control and Prevention, Fort Collins, Colorado, USA (R.S. Nasci, L.R. Petersen);; Emory University School of Medicine, Atlanta (J.M. Hughes);; Oklahoma State Department of Health, Oklahoma City, Oklahoma, USA (K. Bradley);; National Association of County and City Health Officials, Washington, DC, USA (P. Etkind, L. Kan)

**Keywords:** arboviruses, viruses, mosquito-borne encephalitis, West Nile virus, surveillance, health departments, capacity building, vector-borne infections, United States

## Abstract

Before 1999, the United States had no appropriated funding for arboviral surveillance, and many states conducted no such surveillance. After emergence of West Nile virus (WNV), federal funding was distributed to state and selected local health departments to build WNV surveillance systems. The Council of State and Territorial Epidemiologists conducted assessments of surveillance capacity of resulting systems in 2004 and in 2012; the assessment in 2012 was conducted after a 61% decrease in federal funding. In 2004, nearly all states and assessed local health departments had well-developed animal, mosquito, and human surveillance systems to monitor WNV activity and anticipate outbreaks. In 2012, many health departments had decreased mosquito surveillance and laboratory testing capacity and had no systematic disease-based surveillance for other arboviruses. Arboviral surveillance in many states might no longer be sufficient to rapidly detect and provide information needed to fully respond to WNV outbreaks and other arboviral threats (e.g., dengue, chikungunya).

## Abstract 2

Many states cannot rapidly detect and respond to this virus and other emerging arboviral threats.

Before 1999, there was no appropriated funding in the United States for arboviral surveillance, and many states had no arboviral surveillance systems (*2*). After the emergence of West Nile virus (WNV) in New York, New York, in 1999 (*3*), Congress appropriated annual funding to support WNV surveillance activities in affected states and large cities; funds were awarded to these areas through epidemiology and laboratory capacity (ELC) cooperative agreements from the Centers for Disease Control and Prevention. CDC collaborated with state, local health, and academic partners to develop WNV detection, monitoring, and prevention guidance (*4**,**5*). By 2004, WNV had spread across the continental United States (*6*), and transmission to humans had been documented by multiple routes, including blood transfusions and organ transplantation (*7**–**10*). That year, CDC distributed nearly $24 million to all states and 6 large city/county health departments for WNV surveillance and prevention.

In 2000, CDC established ArboNET, a comprehensive national surveillance data capture platform to monitor WNV patterns. In 2003, CDC expanded ArboNET to include other arboviral diseases. ArboNET relies on a distributed surveillance system, whereby ELC-supported state and local health departments report data weekly on detection of arboviruses in humans, animals, and mosquitoes. CDC posts all data on the Internet with weekly updates (*11*). In 2004, the Council of State and Territorial Epidemiologists (CSTE) conducted a WNV surveillance capacity assessment and found that WNV surveillance programs were in place and well developed in jurisdictions receiving WNV surveillance funding (*12*). CSTE attributed the success of capacity development primarily to availability of federal funds and technical guidance from CDC.

Annual funding for WNV and other arbovirus surveillance distributed through the ELC cooperative agreements has steadily decreased since 2006 to 39% of its 2004 zenith, reaching lows of $9.3 million in 2012 and in 2013 (R.S. Nasci, unpub. data). Concomitantly in 2012, the nation experienced the highest incidence of confirmed WNV neuroinvasive disease since 2003 and the highest number of confirmed deaths (286) for any year thus far (*13*). In addition to the continued challenge of WNV to financially stressed arbovirus surveillance systems, there is the growing threat of other arboviral diseases, such as dengue (*14*), chikungunya (*15**–**17*), and Powassan virus encephalitis (*18*).

In August 2013, CSTE conducted another assessment of state and selected local health departments (LHDs) to measure their current surveillance and staffing capacity for WNV and other arboviruses and compare findings with those from the 2004 assessment (*19*). Its objectives were to describe 1) national capacities for surveillance for WNV and other arboviruses in the 50 states and 6 ELC-funded LHDs in 2012 and changes since 2004; 2) surveillance capacities of LHDs with historically high WNV burdens but no direct federal funding and how they compare with those in ELC-supported LHDs; and 3) the outstanding needs to bring US arbovirus surveillance to full capacity.

## Methods

The assessment tool was developed by a working group that included representatives from CSTE, the Association of State and Territorial Health Officials, the National Association of County and City Health Officials, the Association of Public Health Laboratories, the CDC Division of Vector-Borne Diseases, and Emory University. The working group developed the 2013 survey by modifying the 2004 assessment tool and adding unique questions that reflected new WNV surveillance, prevention, and control guidance (*20*) and assessed specific staffing needs, presence of *Aedes aegypti* mosquitoes, and effect of federal WNV surveillance funding reductions on WNV surveillance activities over the past 5 years.

After pilot studies in 7 states and 4 LHDs, CSTE emailed the final state survey to the 50 state health departments and instructed key respondents to obtain relevant information from laboratory and mosquito surveillance and control staff, and complete the assessment online. The Epi Info Web Survey System was used to collect responses (*21*). CSTE used a similar process for distributing the assessment to 30 large city/county health departments that met at least 1 of 3 criteria: 1) receive supplemental WNV surveillance funding through the ELC grant (n = 6 [Washington, DC; New York, NY; Los Angeles County, CA; Chicago, IL; Houston, TX; and Philadelphia, PA); 2) had at least 100 cumulative reported cases of WNV neuroinvasive disease during 1999–2012 (n = 22, excluding 4 of the ELC-funded LHDs); or 3) had recent local dengue transmission (n = 2).

The 2 assessments were analyzed separately. Frequencies of response to each question were examined in aggregate and by groupings of state health departments on the basis of whether they reported a need for additional staff. LHDs were grouped by whether they received federal WNV surveillance funding, which was referred to as ELC-supported. Additional need to achieve full capacity was based on response to the question, “How many additional FTE (full-time equivalent) staff-persons are needed at the state level in your state to achieve full epidemiology and laboratory capacity to conduct WNV and other mosquito-borne disease surveillance?” Full capacity was defined as 1) ability to complete a standard case report form on every suspected/confirmed mosquito-borne arboviral disease case and report it to ArboNET; 2) ability to test for IgM for all relevant arboviruses (including dengue) on any cerebrospinal fluid (CSF) or serum specimen submitted to the state or city/county laboratory for a suspected case of arboviral disease); and 3) having an environmental surveillance system that includes mosquito surveillance to “routinely monitor arboviral activity in all parts of the jurisdiction in which there is the potential for human outbreaks of arboviral disease based on past experience.”

For staffing-related questions, nonresponses were coded as no staff needed. For all other questions, nonresponses were assumed to be missing responses. Differences of >10% between groups being compared were deemed functionally useful and are highlighted in the results. Data analysis was performed by using Microsoft Excel (Microsoft, Redmond, WA, USA) and Epi Info version 7 (CDC, Atlanta, GA, USA).

## Results

All 50 states (100%), all 6 ELC-supported LHDs (100%), and 15 LHDs without ELC support (62.5%) responded. In 2012, nearly all states (98%) conducted surveillance for human WNV disease; fewer conducted WNV-related surveillance for equine disease (90%), mosquitoes (80%), and avian deaths (39%) (Table 1). Less than 60% of jurisdictions contacted medical specialists (neurologists, critical care, infectious disease) to encourage reporting of suspected WNV cases, and less than one third had an active surveillance component for human surveillance. Although only 80% of states conducted mosquito surveillance, 90% collected information about mosquito surveillance from LHDs in their state, including 86% by mosquito species. Overall, 46 (94%) states had at least some information on mosquito populations, either by collecting it themselves or from LHDs. It took a median of 6 days (range 1.5–17 days) from the date a WNV-positive human specimen was collected for data to be reported to the WNV surveillance program, and a median of 16.5 days (range 4–45 days) from date of onset to date reported to ArboNET.

**Table 1:** States conducting selected West Nile virus surveillance activities, United States, 2004 and 2012*

Surveillance activity	No. responding states (% with activity)		% Difference from 2004 to 2012
	2012	2004	
Human surveillance			
Formal surveillance system	50 (98)	49 (100)	−2
Active surveillance component	49 (29)	49 (47)	−18
Use official case definition	50 (88)	49 (88)	0
Require reporting of encephalitis of unknown etiology	50 (48)	49 (63)	−15
To encourage reporting and to suggest a high index of suspicion, did you contact			
Neurologists	48 (50)	48 (60)	−10
Critical care specialists	48 (48)	49 (57)	−9
Infectious disease specialists	48 (58)	49 (82)	−24
Equine surveillance			
Formal surveillance system	49 (90)	49 (94)	−4
Active surveillance component	44 (5)	46 (24)	−19
Designated public health veterinarian within the agency?			
Yes	50 (76)	49 (82)	−6
Avian surveillance			
Formal avian death surveillance	49 (39)	49 (98)	−59
Active component	19 (10)	48 (44)	−34
Sentinel chicken surveillance	50 (10)	–	NA
Adequate access to wildlife expertise within agency	50 (76)	49 (92)	−16
Mosquito surveillance			
Formal surveillance system	49 (80)	49 (96)	−16
Collect information about mosquito surveillance from LHDs in state? (states only)			
Yes	49 (90)	49 (94)	−4
By species?	43 (86)	45 (80)	+6
Do most LHDs in your state conduct surveillance for (states only)			
Adult mosquitoes	44 (34)	44 (48)	−14
Larval mosquitoes	44 (18)	44 (30)	−11
Adequate access to entomologist in agency or by contract	50 (64)	49 (71)	−7

*–, not asked; NA, not applicable; asked; LHDs, local health departments.

Fewer jurisdictions in 2012 than in 2004 conducted WNV-related surveillance activities, particularly avian deaths (26 states, −59%), active human surveillance (9 states –18%), contact with infectious disease specialists (12 states, −24%), and state-level mosquito surveillance (8 states, −16%). In addition, the percentage of states responding that most LHDs in their state conducted adult mosquito surveillance decreased from 48% to 34% (Table 1). There was a slight improvement in timeliness of reporting, from a median of 7 days to 6 days.

In 2012, 92% of states had some public health laboratory capacity for WNV testing to support human surveillance and 84% to support mosquito surveillance (Table 2). Most (93%) states tested human specimens for IgM and mosquito specimens by using PCR (72%) or culture (13%). Relatively few states tested human specimens by using PCR (13%) or culture (2%). When compared with 2004, many fewer laboratories conducted IgG, PCR and culture tests on human specimens in 2012. Testing methods for mosquitoes did not change greatly.

**Table 2:** States with laboratory capacity to support WNV and other arboviral surveillance activities, United States, 2004 and 2012*

Laboratory capacity	No. responding states (% with activity)		% Difference from 2004 to 2012
	2012	2004	
Overall			
Have some in-state capacity for WNV testing	50 (92)	–	NA
Human surveillance			
Test for IgG	46 (48)	47 (72)	−24
Test for IgM	46 (93)	47 (100)	−7
Test by culture	46 (2)	47 (19)	−17
Test by PCR	46 (13)	47 (49)	−36
Test by PRNT	46 (22)	47 (21)	+1
Test all CSF specimens submitted for WNV for ≥1 other arbovirus	43 (60)	–	NA
Avian surveillance			
Test by culture	46 (4)	47 (13)	−9
Test by PCR	46 (39)	47 (77)	−38
Test IgG or IgM	46 (11)	47 (9)	+2
Test by any of above methods	46 (43)	47 (77)	−34
Mosquito surveillance			
In-state capacity to test mosquitoes (state or local level)	50 (84)	–	NA
Testing for >1 other arbovirus	42 (81)	–	NA
Culture or PCR	42 (81)	47 (81)	0
Vec Test or RAMP	42 (19)	47 (21)	−2

*WNV, West Nile virus; –, not asked; NA, not applicable; PRNT, plaque reduction neutralization test; CSF, cerebrospinal fluid; Vec Test, vector test; RAMP, rapid analyte measurement platform.

We also assessed state public health laboratory capacity to test for 10 arboviruses, in addition to WNV, in human serum or CSF specimens. St. Louis encephalitis (SLE) virus testing capacity was most common (34 laboratories), followed by testing for eastern equine encephalitis (EEE) (24), western equine encephalitis (WEE) (16), LaCrosse (16), dengue (9), Powassan (4), chikungunya (2), Colorado tick fever (2), yellow fever (2), and Japanese encephalitis (1) viruses. These laboratories reported performing 41,159 tests for arboviruses in 2012, of which 19,180 (46.6%) were for WNV. Of these tests, the highest percentage of positive test results was for dengue virus (137/328, 41.8%), followed by WNV (2,953/19,178, 15.4%), Powassan virus (62/1,257, 4.9%), LaCrosse virus (121/3,372, 3.6%), SLE virus (164/8,216, 2.0%), Colorado tick fever virus (2/139, 1.4%), and WEE virus (12/3,888, 0.03%). 

Although many laboratories had the capability to test for arboviruses other than WNV, not all routinely did so. Overall, 26 (60%) of 43 responding laboratories reported routinely testing human CSF specimens submitted for WNV for at least 1 other arbovirus. Of these 26 laboratories, 24 routinely tested for SLE virus, 12 for EEE virus, 6 for WEE virus, 5 for LaCrosse virus, and 2 for Powassan viruse. Among laboratories serving the 45 states that either test mosquitoes or use another laboratory, 24 reported routinely testing mosquito pools for SLE virus, 22 for EEE virus, and 13 for the California serogroup. To manage federal WNV surveillance funding reductions over the past 5 years, 57% of states reported eliminating avian death surveillance, 58% decreased mosquito trapping, 68% decreased mosquito testing, and 46% decreased the number of human specimens tested for WNV.

The responses from the 6 LHDs with ELC WNV surveillance support to each surveillance capacity were similar to those from the 50 states in 2004 and 2012, except for laboratory capacity. Currently, only 4 ELC-supported LHDs do some of their own WNV testing.

### Arboviral Surveillance in LHDs without Federal WNV Surveillance Support

The 15 LHDs without ELC grants included 13 with high WNV burden and 2 with recent dengue transmission. These LHDs were generally less likely to take an active role in surveillance for human disease or avian deaths than the 6 LHDs with ELC WNV surveillance support (Table 3). Furthermore, they were less likely to conduct their own mosquito surveillance (67% vs. 100%); 5 LHDs did not conduct any mosquito surveillance. LHDs that conducted mosquito surveillance tended to more consistently conduct larval surveillance and identify trapped mosquitoes to species.

**Table 3:** Local health departments conducting selected WNV surveillance activities, by whether they received federal WNV surveillance funding (ELC) support, United States, 2012*

Surveillance activity	No. responding LHDs (% with activity)		% Difference between no ELC and some ELC support
	No ELC support	ELC support	
Human surveillance			
Formal local-level surveillance system	15 (0)	6 (100)	−100
To encourage reporting and suggest a high index of suspicion, did you contact			
Neurologists	15 (33)	6 (83)	−50
Critical care specialists	15 (47)	6 (83)	−36
Infectious disease specialists	15 (47)	6 (100)	−53
Emergency departments	15 (53)	6 (100)	−47
Equine surveillance			
Formal surveillance system	15 (33)	55 (39)	−6
Designated public health veterinarian within the agency?			
Yes	15 (33)	6 (50)	−17
Avian surveillance			
Formal avian death surveillance	15 (20)	6 (67)	−47
Mosquito surveillance			
Formal surveillance system	15 (67)	6 (100)	−33
For larval mosquitoes?	10 (90)	3 (67)	+23
For adult mosquitoes?	10 (100)	6 (100)	0
Identify trapped mosquitoes to species?	10 (90)	6 (83)	+7
Calculate minimal mosquito infection rates?	10 (50)	6 (83)	−33
Adequate access to entomologist in agency or by contract	14 (31)	6 (50)	−19

*WNV, West Nile virus: ELC, epidemiology and laboratory capacity (received specific WNV surveillance funding through the Epidemiology and Laboratory Capacity Cooperative Agreement); LHDs, local health departments.

Few of these 15 LHDs had their own laboratory capacity to support either testing of human specimens (n = 1) or mosquitoes (n = 3) for WNV. Most were dependent on their state health department for this function.

### Staffing Levels and Need for Additional Staffing

A total of 503 persons worked on arbovirus surveillance in state health departments in 2012. Of these, 206 worked at least half-time on it and 297 worked less than half-time. Overall, 40% of those working at least half-time were CDC funded. When converted to FTEs, there were 208.9 FTEs working on arbovirus surveillance in state health departments in 2012; 17% were epidemiologists, 31% laboratory workers, 27% mosquito surveillance staff, and 25% support staff (Table 4).

**Table 4:** FTE positions for arbovirus surveillance in 2012 and additional FTEs needed by functional job category, 50 states and 21 local health departments, United States*

Characteristic	FTE epidemiologists	FTE laboratory staff	FTE mosquito surveillance staff	FTE support and administrative staff	Total FTEs
State					
2012	34.6 (16.6)	64.6 (30.9)	57.2 (27.4)	52.5 (25.1)	208.9
No. needed	25.1 (20.5)	26.4 (21.5)	53.6 (43.7)	17.5 (14.3)	122.6
Total	59.7	91.0	110.8	70.0	331.5
Local					
2012	32.8 (19.3)	7.4 (4.4)	93.9 (55.6)	34.8 (20.6)	168.9
No. needed	6.2 (9.7)	7.5 (11.7)	36.3 (56.5)	14.2 (22.1)	64.2
Total	39.0	14.9	130.2	49.0	233.1

*Values are no. (%). FTE, full-time equivalent.

In the 21 LHDs, 187 persons worked on arbovirus surveillance in 2012; a total of 104 worked at least half-time and 83 worked less than half-time on it. Similar to state health departments, only 35% of the at least half-time time staff were CDC funded (either directly or through the state). These persons accounted for 168.9 FTEs: 19% were epidemiologists, 4% laboratory workers, 56% mosquito surveillance staff, and 21% support staff (Table 4). LHDs had the same proportions of FTEs involved in mosquito surveillance (56%), regardless of whether they were ELC-supported.

### Staffing Changes from 2004 to 2012 and Additional Needs

**Figure 1:**
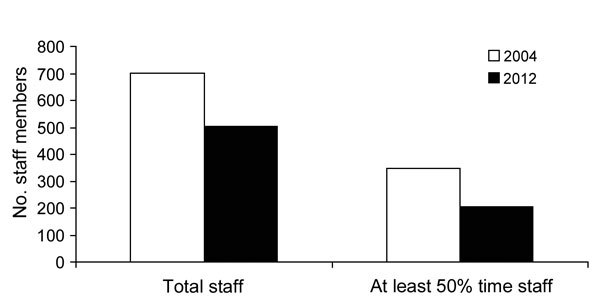
Total and at least 50% time staff performing West Nile virus surveillance in state health departments, United States, 2004 and 2012.

In states and the 6 LHDs with ELC grants for WNV surveillance, the overall numbers of persons working in arbovirus surveillance and the numbers of those working at least half-time on it decreased from 2004 to 2012. In states, the decreases were 28% (from 702 to 503) and 41% (from 348 to 206), respectively (Figure 1). In LHDs, these decreases were 18% (from 228 to 187) and 5% (from 109 to 104), respectively.

Regarding staffing needs, 40 (80%) states reported needing 122.6 additional FTEs, a 59% increase over current capacity: 27 needed epidemiologists, 30 laboratory staff, 28 mosquito surveillance staff, and 19 support staff. Of the 122.6 needed FTEs, the single largest category was mosquito surveillance staff, which accounted for 44% of additional need, followed by laboratorians (22%). For LHDs, 64.2 additional FTEs were needed, a 38% increase, and most (57%) needed positions in mosquito surveillance staff (Table 4).

### Association of Staffing Needs with Level of Arbovirus Surveillance

**Figure 2:**
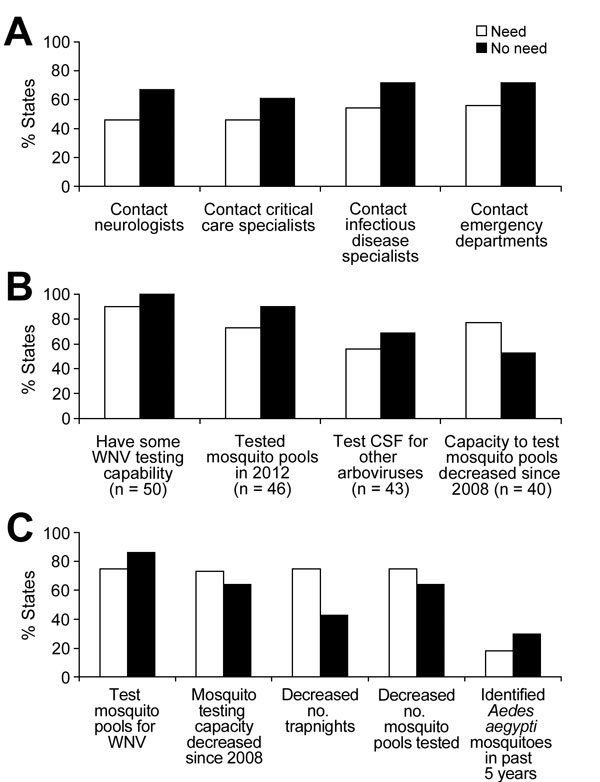
Comparison of surveillance indices in states reporting need for additional staff with those not reporting a need by type of staff needed, United States, 2012. A) Epidemiologists; B) Laboratory staff; C) Mosquito surveillance staff. WNV, West Nile virus; CSF, cerebrospinal fluid. Values in parentheses are number of states.

States needing more staff were less likely to conduct WNV and other arbovirus surveillance activities than those with no need. States needing more epidemiologists were less likely to have conducted outreach to encourage medical specialists to report WNV cases (Figure 2, panel A). These states were also less likely to have performed year-end catch-up surveillance by contacting hospital or commercial laboratories (0% vs. 16%). States reporting a need for laboratorians were less likely to have at least some WNV testing capacity, perform testing on mosquito pools in 2012, and test WNV-positive specimens for other mosquito-borne viruses and were more likely to report a reduction in mosquito pool testing capacity since 2008 (Figure 2, panel B). States needing additional mosquito surveillance staff were less likely to test mosquito pools and to have identified any *Ae*. *aegypti* mosquitoes in the past 5 years and were more likely to have decreased the numbers of mosquito trap-nights and mosquito pools tested and report that their mosquito testing capacity had decreased since 2008 (Figure 2, panel C).

## Discussion

There are several critical objectives of arbovirus-related surveillance at each level of government: 1) monitor for and detect early signs of an outbreak threat to enable a timely response and prevent human illness and death; 2) monitor for arboviruses of human health concern and their vector populations; 3) detect changes in arbovirus disease burden over time and space; and 4) inform the public of the risks and how they can decrease them. Several findings from this assessment highlight the current capacity to meet these objectives and help to inform federal, state, and local public health and preparedness officials interested in evaluating their current arbovirus surveillance capacity.

First, current surveillance capacities at the national and state levels are far greater now than those in 1999, before the introduction of WNV. Almost all states are conducting surveillance for human WNV disease, and most are monitoring mosquito populations for WNV and have some WNV testing capacity. All are reporting WNV and other arbovirus activity to ArboNET.

Second, the ability to detect the early signs of an outbreak of WNV and other arboviruses that can threaten large human populations has been compromised since 2004. Endemic arboviruses that have caused outbreaks of severe illness and death in densely populated areas continue to pose annual threats, and emerging diseases, such as dengue and chikungunya, pose new ones (*14**,**15**–**17**,**22*). Knowledge of local vector mosquito populations and early detection of arbovirus activity in these vectors, animals, and humans are essential to guide public health action ranging from health advisories to mosquito control. Many fewer states now conduct any form of active surveillance that enables rapid detection of the first sentinel human cases of arbovirus disease. Most states have cut back on support for mosquito surveillance. Some states and large metropolitan areas, including some with previously large WNV outbreaks, lack the necessary mosquito surveillance information to anticipate a surge in WNV infection. Most lack the resources to map the distribution and size of either *Ae. aegypti* or *Ae*. *albopictus* mosquito populations to enable risk evaluation or to mount an effective response to identification of local transmission of dengue or chikungunya viruses.

Third, in addition to the decreased ability to monitor vector mosquito populations, testing for arboviruses other than WNV, SLE virus, and EEE virus is patchy and inadequate to detect or monitor their presence in many states. Some endemic arboviruses that cause either encephalitis or acute systemic or febrile disease (e.g., Powassan, LaCrosse, Colorado tick fever, and Heartland viruses) have not been included in systematic public health surveillance, and their ecology and epidemiology might be changing.

For example, Powassan virus spreads to humans from animal reservoirs by the same tick genus (*Ixodes*) that transmits Lyme disease and babesiosis. Although Lyme disease and babesiosis have increased dramatically in incidence and geographic distribution in the United States in the past decades, there is still a poor understanding of Powassan virus epidemiology >50 years after its discovery. Most state health department laboratories do not test for Powassan virus when they test for WNV or other arboviruses, and few clinicians order commercial tests specifically for this virus. Powassan, LaCrosse, and Colorado tick fever viruses were tested for in only 8%, 32%, and 4% of state laboratories, respectively, in 2012. However, a higher percentage of specimens were positive for Powassan and LaCrosse virus infections than for SLE and for Colorado tick fever than EEE or WEE. These results support surveillance for these viruses in jurisdictions with relevant vectors when routinely testing for WNV. If their epidemiology were better understood, estimates of their disease burden could be improved, and the public could be better informed of the risk for infection.

Fourth, state laboratory capacity is essential to enable LHDs to monitor virus activity through mosquito, avian death, or sentinel-chicken surveillance. The ability of ArboNET to synthesize and report useful surveillance information is possible only because of efforts made at each state and local health department to conduct the nationally recommended level of surveillance to meet surveillance objectives. This assessment documents, that as resources have decreased, LHDs dependent on state laboratories to conduct testing for them have reduced or eliminated mosquito-based surveillance to the point where 15% of states no longer provide support for LHDs and one third of responding LHDs in areas with a high incidence of WNV no longer conduct mosquito-based surveillance.

In 2004, all states approached full capacity for 2 of the 3 criteria for full arbovirus surveillance capacity used in this report: ability to complete a standard case report form on every suspected/confirmed mosquito-borne arboviral disease case and report it to ArboNET and having an environmental surveillance system that includes mosquito surveillance “to routinely monitor arboviral activity in all parts of the jurisdiction in which there is the potential for human outbreaks of arboviral disease based on past experience.” In 2012, although the first criterion continued to be met, the second criteria was no longer met. Although the 2004 assessment did not measure the ability to test for IgM for all relevant arboviruses (including dengue viruses) on any CSF or serum specimen submitted to the state or city/county laboratory on a suspected case of arboviral disease, this assessment found that many states are not meeting this remaining criterion.

This assessment has several major limitations. First, not all jurisdictions answered all questions. Second, additional personnel needs were based on state and local health department self-assessment and are subjective. In addition, because of the way the assessment was worded and responded to, we assumed states not specifying a need for additional personnel had no need. Thus, results showing that states that identified a need also performed far fewer surveillance activities than states with no reported additional need are subject to possible inaccuracies in this assumption. Third, the relative role of different surveillance methods shifted between 2004 and 2012. Whereas needs for human surveillance and laboratory testing capability and capacity are largely unchanged, the need for avian death and equine surveillance data in many jurisdictions has decreased, but the need for mosquito surveillance data has increased. Jurisdictions have adjusted resources to accommodate these changes. This adjustment may explain, in part, the generally high US WNV surveillance capacity, despite federal funding cuts of more than 50%. Fourth, measures of workload and staffing need may be difficult to compare among years because they depend, in part, on levels of WNV activity. The human WNV burden in 2012 was more than double that in 2004, which may have influenced estimates of need. Finally, the 2012 assessment did not solicit information on funding or unmet needs for anything other than staff. For example, limited fiscal resources might preclude purchase of updated laboratory equipment and testing reagents, thereby limiting laboratory testing of mosquito pools and testing of human and nonhuman specimens for arboviruses other than WNV. Unmet nonpersonnel needs might have contributed to loss of arbovirus surveillance capacity and would need to be addressed in any effort to maintain or improve it.

In summary, WNV emergence in the United States stimulated building of a robust national arbovirus surveillance system with human and vector early detection components and laboratory services. This system, although still highly functional, has become less robust and might be near a large-scale tipping point, especially in areas of vector surveillance and laboratory support for human diagnostic and mosquito testing. Already, arboviral surveillance is inadequate in many states to rapidly detect and control outbreaks and to give the public the critical information it needs for prevention.
